# Supplementary material for: Polyethylene Glycol Priming Enhances the Seed Germination and Seedling Growth of Scutellaria baicalensis Georgi under Salt Stress
Source: Plants (Basel). 2024 Feb 20;13(5):565. doi: 10.3390/plants13050565 (PMC10934133; doi:10.3390/plants13050565)
Supplement: Supplementary file 1 [file plants-13-00565-s001.zip › plants-2818805-supplementary.pdf]

Table S1. Primers used for qRT-PCR analysis.

Gene	Primer Sequence	
	**Forward(5′-3′)**	**Reverse(5′-3′)**
SbSOD1	AGTCCTCCCTTTCGTTCC	ACCGTTCTGGGTTTGTTG
SbSOD2	CTCACCCAGGAGGACGAT	GCAACTATGTTTCCCAGGTC
SbAPX	TACGCCAAGAGGATAGCA	GGTAAATCGTCTGGGAAG
SbPOD1	TCAAACCTACCACCACCT	GTATGCCAAATCCAGAGT
SbPOD2	GGACTGTAAGGCTGGGAAGA	GACTAAATCAAGTGGTGCTAAA
SbPOD3	GCAGCCAACACCCAAATC	GCACCTCGCCTGTCCTAT
SbActin	TTGATCTTGCTGGTCGTGATCTCA	TGTTTCTAGCTCTTGCTCGTAGTCG
